# RESISTANCE EXERCISE AND BRAIN CONNECTOME IN MILD COGNITIVE IMPAIRMENT

**DOI:** 10.1002/alz.092342

**Published:** 2025-01-09

**Authors:** Isadora Cristina Ribeiro, Brunno de Campos, Camila Vieira de Ligo Teixeira, Marco Carlos Uchida, Ítalo Karmann Aventurato, Marjorie Cristina Rocha da SIlva, Brenda Costa Gonçalves, Liara Rizzi, Ana Luiza Gonçalves Rochetti, Emanuel R. Guimaraes, Thamires Naela Cardoso Magalhães, Paula Fernandes, Marcio Luiz Figueredo Balthazar

**Affiliations:** ^1^ Universidade Estadual de Campinas (UNICAMP), Campinas, SP Brazil; ^2^ Rockefeller Neuroscience Institute, West Virginia University, Morgantown, WV USA; ^3^ University of Campinas, Campinas, SP Brazil; ^4^ UNICAMP ‐ UNIVERSIDADE ESTADUAL DE CAMPINAS, CAMPINAS, SP Brazil; ^5^ Memory and Aging Center, UCSF Weill Institute for Neurosciences, University of California, San Francisco, San Francisco, CA USA; ^6^ Texas A&M University, College Station, TX USA; ^7^ University of Campinas (Unicamp), Campinas, SP Brazil

## Abstract

**Background:**

Individuals with Mild Cognitive Impairment (MCI) are at greater risk of developing Alzheimer's disease (AD). Previous studies have shown that physical exercise is a protective factor against the clinical evolution of dementia in MCI. Lower muscle strength levels are associated with a greater risk of AD incidence. Physical exercises can also promote improvements in brain networks' functional connectivity (FC). However, the influence of resistance exercise, which significantly impacts the development of muscular strength and cognition, remains unknown concerning FC in MCI. We aimed to investigate the FC of brain networks after 24 weeks of resistance training.

**Method:**

37 older adults with MCI were investigated. Nineteen performed the resistance training protocol, and 18 constituted the control group, not performing the exercises. Participants were evaluated before and after the intervention using a magnetic resonance imaging device (3 Tesla) for functional magnetic resonance imaging. FC was assessed in Matlab software using the uf2c program to investigate intra‐network connectivity, considering a p‐value of 0.05 with an FDR‐corrected comparison. We evaluated 12 networks: AnteSalience, PostSalience, Auditory, BasalGanglia, Dorsal Default Mode Network, Ventral Default Mode Network, Language, Left executive control network, Right executive control network, Sensorimotor, Visual and Visuospatial networks.

**Result:**

AnteSalience connectivity decreased in the control group (T‐score > 3.1473) and increased in the exercise group in the PostSalience (T‐score > 3.7114). The results suggest that the training intervention also increased the FC of the visuospatial network (uncorrected results T‐score > 2.7195), while the control group showed no changes. None of the other networks shows differences between the pre‐and post‐intervention moments. The intervention time may have influenced the results, as changes in FC tend to occur over extended periods; even so, resistance exercise proved influential.

**Conclusion:**

FC increased or tended to increase in some networks in the exercise intervention group, while in the control group, it decreased or remained stable, suggesting that exercise may be a beneficial modulator of brain connectivity in specific networks such as AnteSalience, PostSalience, and the Visuospatial network. More studies are suggested, especially those that monitor FC changes over extended periods.

